# Roles of nucleic acid substrates and cofactors in the vhs protein activity of pseudorabies virus

**DOI:** 10.1186/s13567-015-0284-y

**Published:** 2015-12-24

**Authors:** Ya-Fen Liu, Pei-Yun Tsai, Fong-Yuan Lin, Kuan-Hsun Lin, Tien-Jye Chang, Hui-Wen Lin, Songkhla Chulakasian, Wei-Li Hsu

**Affiliations:** Graduate Institute of Microbiology and Public Health, College of Veterinary Medicine, National Chung Hsing University, 250 Kou Kuang Road, Taichung, 402 Taiwan; Department of Beauty Science, MeiHo University, Neipu, Pingtung County Taiwan; Department of Veterinary Medicine, College of Veterinary Medicine, National Chung Hsing University, Taichung, Taiwan

## Abstract

Pseudorabies virus (PrV) belongs to the *α*-*herpesvirinae* of which human simplex virus (HSV) is the prototype virus. One of the hallmarks of HSV infection is shutoff of protein synthesis that is mediated by various viral proteins including vhs (virion host shutoff), which is encoded by the UL41 gene. However, the function of PrV vhs is poorly understood. Due to the low sequence similarity (39.3%) between the HSV and PrV UL41 proteins, vhs might not share the same biochemistry characteristics. The purpose of this study was to characterize the nuclease activity of the PrV vhs protein with respect to substrate specificity, its requirements in terms of cofactors, and the protein regions, as well as key amino acids, which contribute to vhs activity. Our results indicated that, similar to HSV vhs, PrV vhs is able to degrade ssRNA and mRNA. However, PrV vhs also targeted rRNA for degradation, which is novel compared to the HSV-1 vhs. Activity assays indicated that Mg^2+^ alone enhances RNA degradation mediated by PrV vhs, while K^+^ and ATP are not sufficient to induce activity. Finally, we demonstrated that each of the four highly conserved functional boxes of PrV vhs contributes to RNA degradation and that, in particular, residues 152, 169, 171, 172, 173 343, 345, 352 and 356, which are conserved among *α*-*herpesviruses*, are key amino acids needed for PrV vhs ribonuclease activity.

## Introduction

Pseudorabies virus (PrV), an enveloped dsDNA virus belonging to the *α*-*herpesvirinae*, causes Aujeszky’s disease of swine [1]. Similar to the herpes simplex virus type 1 (HSV-1), the prototype virus of *α*-*herpesviruses*, the PrV tegument contains the virion host shutoff (vhs) protein, which is encoded by the UL41 gene [2]. HSV vhs has ribonuclease activity that produces a reduction in overall protein synthesis via mRNA degradation; this effect on the cellular translational apparatus is beneficial in terms of viral protein synthesis [3]. In addition to cellular mRNA, vhs also degrades viral RNAs, which facilitates a sharpening of the transitions between the successive phases of viral protein synthesis [3]. Moreover, in a mouse model, replication of a vhs-deficient HSV-1 was found to be significantly attenuated and this was correlated with the degradation of cytokine mRNAs, including type I IFN and IL-8 [4]. It has also been reported that vhs is responsible for a reduction in components of major histocompatibility complex class I in cells infected with HSV-2. These findings indicate that vhs-dependent ribonuclease activity is important to HSV virulence [5].

The mechanism of vhs-induced ribonuclease activity by HSV-1 has been intensively studied. Initially, the ribonuclease activity of vhs was identified in HSV-1infected cells [3], and its substrate specificity was further characterized using an in vitro assay system that consisted of HSV-1 infected cell lysate [3, 6, 7]. Subsequently, the protein’s endonuclease activity was explored using vhs protein that had been translated using a variety of different systems, including rabbit reticulocyte lysate (RRL) [8] and the budding yeast *Saccharomyces cerevisiae* [9]. Vhs translated using RRL induced degradation of a variety of RNAs including ssRNA and mRNA with a 5′ cap and a 3′poly (A) tail [8]. Furthermore, it was demonstrated that vhs preferentially targets regions adjacent to the translation initiation sites of mRNAs, for example the 5′ end of the RNA [10], or the 3′-flanking sequences of the internal ribosome entry sites (IRES) of encephalomyocarditis virus (EMCV) and poliovirus [8]. Moreover, mammalian cellular factors were found to increase overall RNase activity, as well as inducing the preferential cleavage of HSV-1 vhs produced using the *Saccharomyces cerevisiae* system at particular sites [9]. It was further demonstrated by other research groups that there was an association of vhs with components of the cap-binding complex eIF4F, including eIF4H, eIF4A, and eIF4B; this was found to be responsible for the specific targeting pattern of vhs [11–13].

In 1991, using a cytoplasmic extracts from HSV-1-infected HeLa cells, Krikorian and Read revealed that vhs-mediated RNase activity was not inhibited by RNase inhibitor; however it was found to be strongly dependent on presence of divalent cations such as Mg^2+^, but did nor require energy components such as ATP or GTP [7]. However, it was influenced by K^+^; efficient vhs-induced degradation of RNA occurs at the concentration of K^+^ up to 200 mM, but not when the concentration is higher than 500 mM. The requirement of Mg^2+^ was also confirmed using an assay based on vhs translated by RRL [8]. Although ATP is not essential for vhs activity, it is required for optimal vhs mediated RNA degradation in the presence of Mg^2+^.

HSV-1 vhs targets mRNA, but spares rRNA and tRNA from degradation [3, 6, 7]. Sequence analysis has indicated that vhs shares similarity with RNase H, which is able to degrade ssRNA from DNA/RNA hybrids, and with cellular nucleases of the FEN-1 family that shows exo/endonuclease activity with RNA and DNA substrates [14]. However, the RNase activity of vhs, in general, has been tested only using ssRNA, and whether vhs has bioactivity, particularly substrate specificity resembling RNase H or FEN-1, remains unclear.

**Figure 1:**
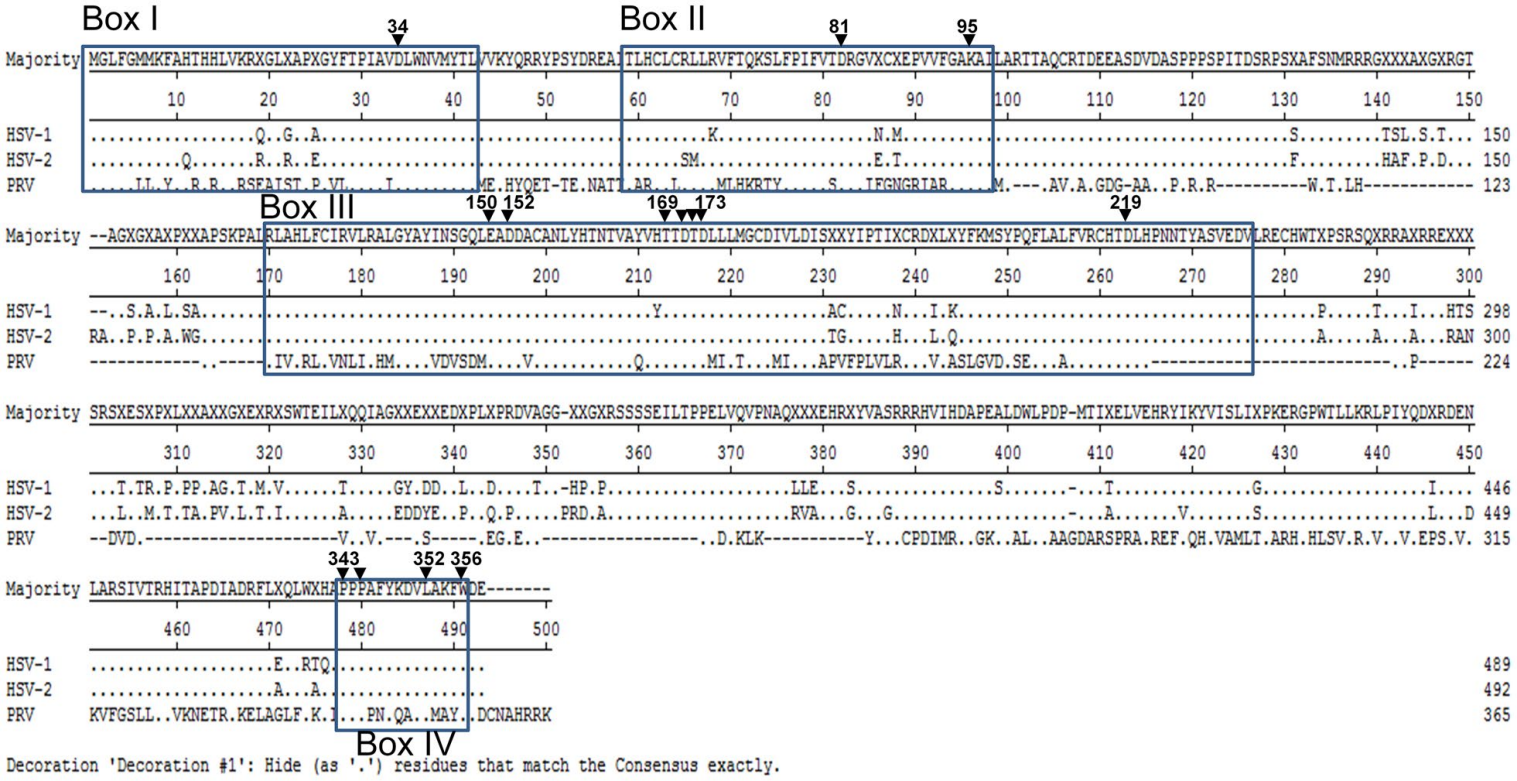
**Sequence alignment of the vhs coding regions.** The vhs sequences of HSV-1, HSV-2, and PRV were analysed by DNA Star MegaAlign software. Identical residues are denoted as “.” and deleted amino acids are indicate as “-”. Four conserved region, designated as Boxes I–IV (reported in Berthomme et al. [15]) are marked. In addition, several deduced residues, numbered according to the PrV vhs gene, that have been reported as being responsible for ribonuclease activities are indicated with arrowheads.

Based on sequence alignments analysis, the vhs protein has been found to be conserved across many members of *α*-*herpesviruses*, including, HSV-1, HSV-2, varicella zoster virus (VZV), PrV, and equine herpesvirus (EHV-1) [15]. Notably, there are four deduced functional regions, namely Boxes I–IV, present in PrV vhs (Figure 1). Lin et al. demonstrated that PrV vhs is involved in virion host shut off of protein synthesis and also contributes to PrV virulence in a mouse model [13]. In addition, the ribonuclease activity of PrV vhs was initially identified using an in vitro system where the purified recombinant vhs protein was fused with thioredoxin and found to be able to degrade ssRNA markers [16]. However, up to the present, the mechanism of action of vhs remains largely unknown. Taking into consideration the low similarity found when the vhs protein sequences of PrV and HSV-1 are compared (39.3%), these two vhs orthologs might not share the same characteristics. Thus the biochemical characteristics of PrV vhs, including the enzyme’s substrate specificity, its requirements in terms of catalytic factors (Mg^2+^, K^+^ or ATP), the types of nucleic acid it prefers to degrade and the key residues involved in the vhs-dependent ribonuclease activity, were explored in this study using systems similar to those described by other research groups [9, 17, 18].

## Materials and methods

### Cell culture

Human embryonic kidney 293T (HEK293T) cells was propagated in Dulbecco’s Modified Eagle Medium (DMEM, Gibco BRL, Life Technologies Corporation, Carlsbad, CA, USA) containing 10% bovine fetal calf serum (Hyclone, Logan, UT, USA) and 1% of penicillin–streptomycin (Gibco, BRL). Cells were cultured at 37 °C in a 5% CO_2_ atmosphere.

### Sequence alignment of the vhs proteins of α-herpesviruses and other ribonuclease

The amino acid sequences of HSV-1 (accession number CAA96525), HSV-2 (AEV91380.1), and PrV (AID18742.1) vhs were aligned by the DNASTAR Software MegAlign (DNASTAR, Inc. Madison, Wisconsin, USA).

### Construction of plasmids expressing various vhs proteins

**Figure 2:**
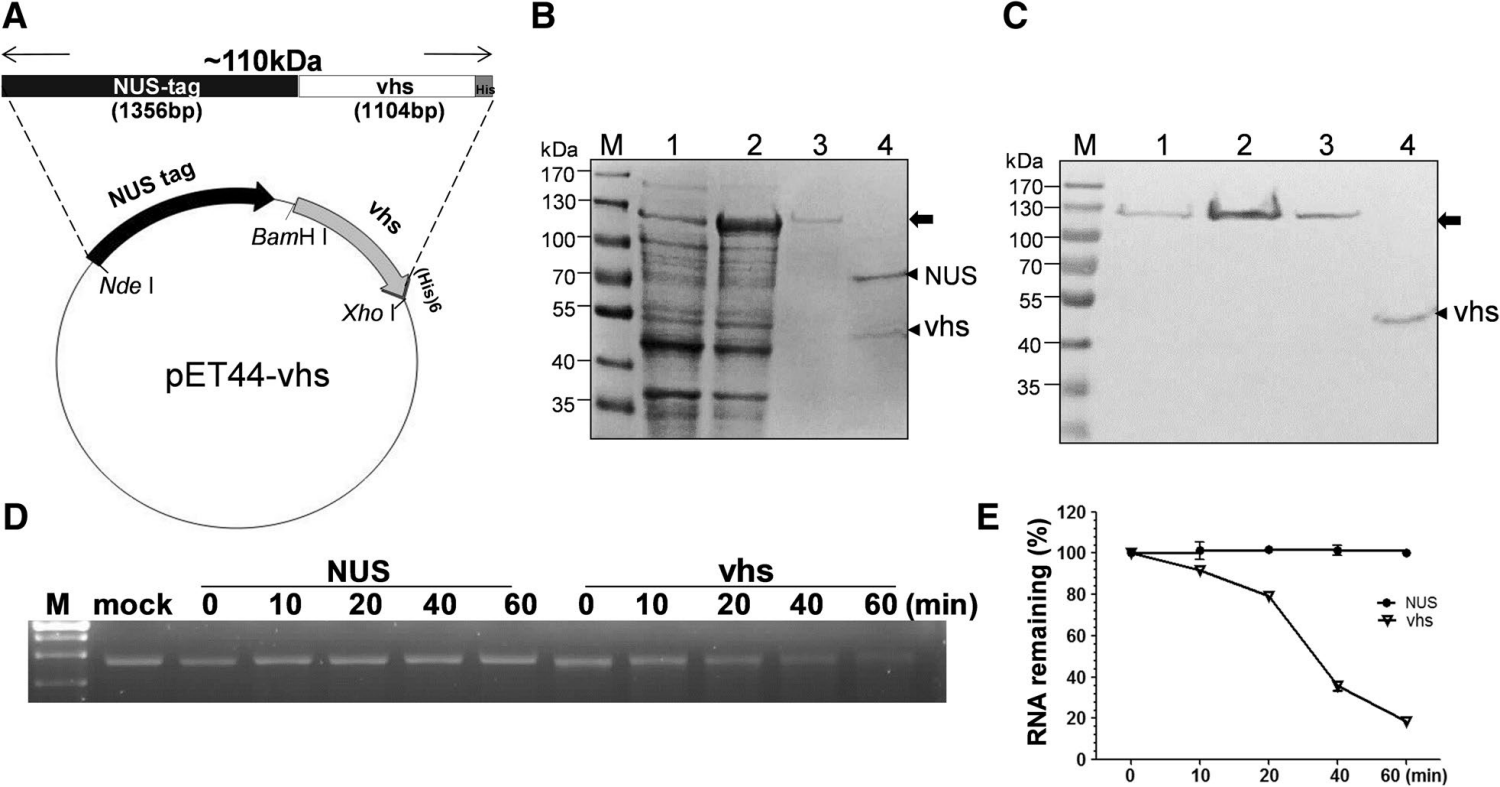
**Expression and purification of recombinant PrV vhs protein using** ***E. coli*** **.** The coding region of PrV vhs was inserted downstream of the NUS gene in pET44 vector (**A**). In this expression system, the recombinant vhs protein was fused with NUS tag protein and a his tag at the N and C terminus, respectively. Under IPTG induction, NUS-vhs-his with a predicted molecular weight of ~110 kDa was expressed (**B**, lane 2) and then purified using Ni–NTA beads (lane 3). Subsequently, the N-terminal NUS tag was removed by thrombin treatment (lane 4). M protein marker; Lane 1 non-induction lysate. The identity of the PrV vhs was initially confirmed by Western blot analysis using antibodies against the his tag (**C**). The arrow indicates the uncleaved NUS-vhs-his recombinant protein, and the thin arrowheads indicate the NUS tag and vhs-his after thrombin digestion. The ribonuclease activity of PrV vhs was tested using single stranded RNA (**D**). RNA substrates generated by in vitro transcription were incubated with assay buffer (mock), NUS protein (as a negative control), or recombinant PrV vhs protein for the indicated times (0, 10, 20, 40, and 60 min). The RNA reaction products were then analyzed by 1.3% agarose-formaldehyde gel electrophoresis and the amount of RNA remaining was measured by imageJ system. The experiment was repeated three times and the RNA content at the time point of 0 h was set to 100% (**E**).

To generate the recombinant vhs proteins for in vitro assay, wild type PrV vhs coding region was constructed in vector pET44a (+) (Novagen, Germany) in two steps. Initially, *nus* fragment with deletion of the two hexa-histidine (6-His) sequences in front and rear ends of nus-coding region was amplified from pET44a (+) by polymerase chain reaction (PCR) using the primer sets (*Nde*I-Nus-F: GGCATATGAACAAAGAAATTTTGGCTGT and *Sac*II-Nus-R: ACCCGCGGACGCTTCGTCACCGAACCAGCA). For the ease of further cloning, all the primer sets contain sequences of restriction enzyme sites, shown as underlined. The thermal cycling conditions were: 94 °C (5 min) followed by 35 cycles of denaturation (94 °C, 1 min), annealing (55 °C, 1 min), and extension (72 °C, 2 min), and finished with a final extension (72 °C, 7 min). The PCR product with expected size of 1.6 kilo base pairs (kb) was purified and digested with *Nde*I and *Sac*II restriction enzymes. Modified *nus* fragment was then cloned into vector pET44a (+) to replace the original 6-His *nus* sequences; the resulting plasmid was named pET44a (∆his-tag). Subsequently, PrV vhs gene amplified from the genome of PrV TNL strain [19] by PCR using the primer sets (PrV-F: AAGGATCCGCCATGGGGCTCTTTGGCCTTT and PrV-R: AACTCGAGTTATTTTCTCCTGTGGG) was inserted into pET44a (∆his-tag) vector linearlied with *Bam*HI and *Xho*I; the resulting plasmid was named pET44-PrV vhs. The plasmid map is presented in Figure 2A.

To characterize the function of vhs protein in mammalian cells, eukaryotic plasmids expressing vhs proteins of PrV or HSV-1 were constructed. Initially, HSV-1 vhs coding region was amplified by PCR using the primer set (HSV1-F: AAAAAGCTTATGGGTTTGTTCGGGATGAT and HSV1-R: AACTCGAGCTACTCGTCCCAGAATTTGGCC) from HSV-1 KOS strain (a gift from Professor S. H. Chen, Department of Microbiology and Immunology of National Cheng Kung University, Taiwan). The resulting PCR product (1.5 kb) was digested with *Hin*dIII and *Xho*I restriction enzymes, which are present in the primers, and then was subcloned into the expression vector pcDNA3.1(+) (Invitrogen, Carlsbad, CA, USA). As for PrV vhs, the vhs fragment (1.1 kb) obtained from pET44-PrV vhs plasmid digested with *Bam*HI and *Xho*I restriction enzymes was subcloned into vector pcDNA3.1(+). Plasmid expressing HSV-1 vhs and PrV vhs were designated as pcDNA3.1/HSV vhs, pcDNA3.1/PrV vhs, respectively.

In addition, a series of plasmids bearing PrV vhs mutations were generated. Based on the sequence alignment, four highly conserved motifs (Box I–IV) and several key residues contributed to vhs mediated shutoff of protein synthesis among α-herpesviruses were deducted (Figure 1). To evaluate the contribution of each individual functional motifs, PrV vhs fragment with deletion of one of the four deduced boxes (∆Box I–IV) was generated by PCR. ∆Box I of vhs fragment was amplified by PCR using Pfu DNA polymerase (NEB, NEW ENGLAND BioLabs^®^ INC.) and primer sets with built-in sequences of *Bam*HI/*Xho*I as shown in underlines (Box 1–F: AAGGATCCGCCATGGAGAAGCACTAC and PrV-R: AACTCGAGTTATTTTCTCCTGTGGG). The PCR condition were 94 °C (7 min) followed by 35 cycles of denaturation (94 °C, 45 s), annealing (55 °C, 50 s), and extension 68 °C (1 min 30 s), and finished with a final extension (68 °C, 7 min). ∆Box II or ∆Box III of vhs fragment were generated using a PCR-driven overlap extension method [20]. First, to amplify the 5′ arm sequence of ∆Box II vhs by primer PrV-F (AAGGATCCGCCATGGGGCTCTTTGGCCTTT) and Box2-R (CATGATGGCCTTGGCCGTGGTCGCGTTGTC). In addition, 3′ arm of PrV vhs Box II was amplified with primer Box2-F (GACAACGCGACCACGGCCAAGGCCATCATG) and PrV-R. Subsequently, these two PCR fragments was served as template for the sequel PCR amplification using outer primer set (PrV-F/PrV-R) for amplifying ∆Box II vhs fragment. Similarly, ∆Box III of vhs fragment was amplified by this 2-step PCR with primer sets (PrV-F/Box3-R (CTCCAGCGACTCGGCCGGCGCGTGCAGCAT), Box3-F (ATGCTGCACGCGCCGGCCGAGTCGCTGGAG)/PrV-R, and the resulting amplicons were then used as template for the second run PCR using primer set PrV-F/PrV-R. The Box IV is located at very 3′-end of PrV vhs gene; ∆Box IV of vhs fragment was generated by PCR using forward primer PrV-F and a degenerated reverse primer Box4-R (AACTCGAGTTATTTTCTCCTGTGGGCGTTACAGTCGTCTATGTGCTTCCAAAAC) containing the remnant 3′ termini of vhs sequences. All the products were digested with two enzymes (*Bam*HI and *Xho*I) and then cloned into pcDNA3.1(+) vector linearlized with the same enzymes. The authenticity of the plasmids (pcDNA3.1/PrV vhs ∆Box I, II, III, IV) was determined by automatic sequencing (Mission Biotech, Taipei, Taiwan).

In addition, the QuikChange™ site-directed mutagenesis system (Stratagene, California, USA) was used to substitute the individual fourteen conserved residues (at amino acid 34, 81, 95, 150, 152, 169, 171, 172, 173, 219, 343, 345, 352 and 356) to Alanine following the manufacturer’s procedure. The sequence of mutagenic primers was listed in Table 1. The PCR amplification conditions were 95 °C for 5 min followed by 18 cycles of denaturation at 95 °C for 30 s, annealing at 60 °C for 1 min, DNA extension at 68 °C for 8 min and a final extension at 68 °C for 7 min. All amplification cycles were performed in a DNA thermal cycle (GeneAmp PCR system 2700) and the PCR product was then treated with *Dpn* I, followed by transformation into the XL1-Blue competent cells. The plasmid with expected mutation sequences was determined by automated DNA Sequencing (Mission Biotech, Taipei, Taiwan).

**Table 1:** Oligonucleotides used for construction of PrV vhs bearing point mutations in this study.

	Oligonucleotides containing altered codons
D34-A(GAC-GCC)	(F) 5′-ACGCCCATCGCCATC***GCC***CTGTGGAACGTCATG-3′ (R) 5′-CATGACGTTCCACAGGGCGATGGCGATGGGCGT-3′
D81-A(GAC-GCC)	(F) 5′-CCCATCTTCGTCTCG***GCC***CGCGGCATCTTCGGG-3′ (R) 5′-CCCGAAGATGCCGCGGGCCGAGACGAAGATGGG-3′
K95-A(AAG-GCG)	(F) 5′-GTCGCGCACGGCGCC***GCG***GCCATCATGGCCGCG-3′ (R) 5′-CGCGGCCATGATGGCCGCGGCGCCGTGCGCGAC-3′
E150-A(GAG-GCG)	(F) 5′-GACGTGTCGGACATG***GCG***GCGGACGACGTCTGC-3′ (R) 5′-GCAGACGTCGTCCGCCGCCATGTCCGACACGTC-3′
D152-A(GAC-GCC)	(F) 5′-TCGGACATGGAGGCG***GCC***GACGTCTGCGCCAAC-3′ (R) 5′-GTTGGCGCAGACGTCGGCCGCCTCCATGTCCGA-3′
D169-A (ACG-GCG)	(F) 5′-GTCGCGCAGGTGCAC***GCG***ACCGACACGGACATG-3′ (R) 5′-CATGTCCGTGTCGGTCGCGTGCACCTGCGCGAC-3′
D171-A(GAC-GCC)	(F) 5′-CAGGTGCACACGACC***GCC***ACGGACATGATCCTC-3′ (R) 5′-GAGGATCATGTCCGTGGCGGTCGTGTGCACCTG-3′
D173-A(GAC-GCC)	(F) 5′-CACACGACCGACACG***GCC***ATGATCCTCACCGGG-3′ (R) 5′-CCCGGTGAGGATCATGGCCGTGTCGGTCGTGTG-3′
D219-A(GAC-GCC)	(F) 5′-GTGCGCTGCCACACG***GCC***CTGCACCGGGCGCCG-3′ (R) 5′-CGGCGCCCGGTGCAGGGCCGTGTGGCAGCGCAC-3′
172(delete ACG)	(F) 5′-GTGCACACGACGGAC**-**GACATGATCCTCACC-3′ (R) 5′-GGTGAGGATCATGTCGTCCGTCGTGTGCAC-3′
343(delete CCC)	(F) 5′-TTTTGGAAACACATA**-**CCGCCCCCGAACTAC-3′ (R) 5′-GTAGTTCGGGGGCGGTATGTGTTTCCAAAA-3′
345(delete CCC)	(F) 5′-AAACACATACCCCCG**-**CCGAACTACCAGGCC-3′ (R) 5′-GGCCTGGTAGTTCGGCGGGGGTATGTGTTT-3′
352(delete TCC)	(F) 5′-AACTACCAGGCCGTC**-**ATGGCGTACTGGGAC-3′ (R) 5′-GTCCCAGTACGCCATGACGGCCTGGTAGTT-3′
356(delete TGG)	(F) 5′-GTCCTCATGGCGTAC**-**GACGACTGTAACGCC-3′ (R) 5′-GGCGTTACAGTCGTCGTACGCCATGAGGAC-3′

### Preparation of purified PrV NUS-vhs recombinant proteins

Plasmids expressing PrV vhs fused with NUS tag protein at N-terminus end and 6-histidine tag at C-terminus end was transformed into *Escherichia coli* (*E. coli*) strain BL21. Expression of all the recombinant proteins was induced by 0.8 mM of isopropyl β-d-1-thiogalactopyranoside (IPTG) and further purified by the chelating Sepharose Fast Flow (GE Healthcare) following the method described in one previous study [21]. Purity of recombinant proteins was initially confirmed by sodium dodecyl sulfate polyacrylamide gel electrophoresis (SDS-PAGE) and the concentration of recombinant proteins was estimated by comparing with the band intensity of known concentration of bovine serum albumin (BSA) quantified by National Institutes of Health ImageJ software version 1.43 [22].

To remove the NUS protein tag from vhs, proteolytic cleavage was conducted using biotinylated thrombin kit (Novagen, Germany) following manufacturer’s instructions. Briefly, 400 μg recombinant proteins was incubated with 0.5 U biotinylated thrombin, 50 μL 10× thrombin cleavage buffer (200 mM Tris–HCl, pH 8.4, 1.5 M NaCl, 25 mM CaCl_2_) at 20 °C for 4 h. After cleavage reaction, biotinylated thrombin was removed by chromatography using streptavidin agarose beads (8 μL), and the flow through solution containing vhs was collected. Finally, the recombinant protein was dialyzed against vhs assay buffer (1.6 mM Tris acetate, 80 mM potassium acetate, 2 mM magnesium acetate, 0.1 mM DTT, 0.25 mM ATP, pH 7.8) at 4 °C to remove the excess imidazole. The protein concentration was estimated by SDS-PAGE using the standard curve of known concentrations of BSA.

### Western blot analysis

Proteins resolved on 10% SDS-PAGE were further transferred to nitrocellulose membrane using Mini Protein III equipment (Bio-Rad Laboratories, Richmond, CA, USA) for Western blot analysis. After blocking with phosphate-buffered saline with tween^®^ 20 (PBST) containing 5% skimmed milk for 1 h at room temperature. Membrane was incubated with 1:2000 diluted anti-His tag antibody (AbD Serotec., Kidlington, UK) in 5% skimmed milk at 4 °C for overnight. After washes with PBS-T (PBS containing 0.1% tween 20) buffer, the filter was then incubated with 1:10 000 diluted secondary antibody, goat anti-mouse IgG conjugated HRP (Jackson ImmunoResearch Laboratories, Inc., West Grove, PA, USA), for 1 h at room temperature. After several times of PBS-T washes to remove the unbound antibodies, the signal was detected by TMB reagent (KPL, Kirkegaard and Perry Laboratories, Inc., Gaithersburg, MD, USA).

### In vitro translation of vhs proteins

DNA (2 μg) of plasmids pcDNA3.1/HSV vhs or pcDNA3.1/PrV vhs were individually added into a 40 μL rabbit reticular lysate (RRL; TNT^®^ T7 Quick coupled Translation system, Promega, Madison, WI, USA) reaction mixture containing 20 μCi of [S^35^] methionine. Translation reactions were carried out at 30 °C for 1.5 h. The translation reaction products were confirmed with 10% SDS-PAGE and autoradiography.

### Preparation of various assay substrates for in vitro vhs assay

Different kinds of nucleic acid substrates, including DNA, RNA, and RNA/DNA hybrid, were used in this study. RNA substrates, including ssRNA, or ssRNA with cap and polyA tail (mRNA), and rRNA were generated as follows:

First of all, the template for transcribing control ss RNA was generated by digestion of SP6LUC plasmid (Promega) with *Xmn* I followed by in vitro transcription by SP6 RNA polymerase. After purifying with phenol/chloroform, RNA were further treated with RNase-free DNase (Promega, Madison, WI, USA) at 30 °C for 1 h to remove DNA template. The template for mRNA transcription was generated from pcDNA3.1-MV-LUC plasmid by PCR using forward primer containing upstream sequences of T7 promoter and the reverse primer containing a 21-nucleotide polyA 3′ overhang [23]. The PCR products were purified and transcribed by T7 RNA polymerase (Promega) in the presence of mono-methylated cap analog m7G5′-pppp-G (Epicentre, Madison, WI, USA). The rRNA was extracted from transfected 293T cells using Trizol^®^ reagent (Invitrogen) following the manufacturer’s instruction. The ssRNA was resolved on a 1.3% formaldehyde denatures agarose gel and the concentration was calculated by spectrophotometer (GE NanoVue).

Several of DNA substrates were prepared for vhs assay, including M13KE phage single stranded DNA (ssDNA) obtained from NEW ENGLAND BioLabs^®^ INC and uncut double stranded DNA, plasmid pET-44a (+). For linearlized dsDNA, pET-44a (+) DNA was treated with restriction enzyme *Bam*HI and *Xho*I and purified.

Finally, to generate DNA/RNA hybrid, firstly, sense ssRNA was transcribed from *Bam*H I-linearized plasmid pcDNA3.1 (−) by T7 RNA polymerase. The runoff transcript (88 nucleotides) was treated with calf intestine phosphatase (NEW ENGLAND BioLabs^®^ INC), and end-labeled with γ-[p32] ATP by T4 polynucleotide kinase PNK enzyme (NEW ENGLAND BioLabs^®^ INC).Then 2 μM of isotope labeled ssRNA and ssDNA oligonucleotide (CGGGCGAATTGGAGCTCCACCGCGGTGGCGGCCGCTCGAGTCTAGAGGGCCCGTTTAAACGCTAGCCAGCTTGGGTCTCCC) synthesized from Mission Biotech Company were mixed in 100 mM NaCl annealing buffer. Sample was heated to 80 °C for 5 min and gradually cooled down to room temperature. The annealed samples was resolved on 8% acrylamide PAGE and double-stranded product was then isolated.

### In vitro vhs assay using isotope labelled ss RNA and RRL translated vhs proteins

Firstly, Renilla Luciferase (R-Luc) RNA fragment was transcribed from p2LUC plasmid that was linearized with restriction enzyme *Bam*H I by T7 RNA polymerase (Promega, USA) with α-[p^32^]-ATP incorporation for 2 h at 37 °C. Then, the 0.9 kb runoff transcript was purified with MicroSpin G-25 columns (GE Healthcare Life science, USA).

For in vitro vhs assay, R-Luc RNA substrates (3 × 10^5^ counts per min) was incubated with RRL translated PrV vhs or only RRL(as a negative control) in vhs assay buffer (80 mM K^+^, 2 mM Mg^2+^, 0.25 mM ATP, 0.1 mM DTT, 1.6 mM Tris–HCl, pH 7.8) at 37 °C. At the indicated time points (0, 15, 30 min), assay products were recovered by RNeasy^®^ mini kit (QIAGEN, Center Mainz, Germany) and separated on a 1.3% formaldehyde agarose gel. All signals were transferred onto a Hybond-N^+^ membrane (GE Healthcare Bio-Science Corp., Piscataway, NJ, USA) and exposed to a phosphoimage screen (Fuji, Tokyo, Japan) and detected with a Bio-Imaging Analyzer (BAS-2500; Fuji).

### In vitro vhs ribonuclease activity assay

Purified recombinant vhs proteins expressed from *E. coli*, or vhs proteins translated in rabbit reticulocyte lysate (RRL) were used in this study. In one reaction, 0.05 μg of ssRNA were incubated with 0.25 μg of NUS protein (as a negative control), or 0.25 μg of purified PrV vhs protein in vhs assay buffer (80 mM K^+^, 2 mM Mg^2+^, 0.25 mM ATP, 0.1 mM ATP, 0.1 mM DTT, 1.6 mM Tris–HCl, pH 7.8) for the indicated time points. RNA reaction products were then analyzed by 1.3% agarose-formaldehyde gel electrophoresis and RNA quantity was measured by imageJ system.

For study the degradation pattern, and also to rule out residual endogenous RNase activity from *E. coli*, vhs translated from RRL was also used for RNA assay following the method described previously [8]. Briefly, RNA substrates were produced and labeled with α-[p^32^] ATP by in vitro transcription and then incubated with assay buffer (mock control), RRL reagent (negative control), or RRL translated vhs at 37 °C for the indicated time points (0, 15, 30 min). RNA products were recovered by RNeasy^®^ mini kit (QIAGEN, Center Mainz, Germany) and analyzed by 1.3% formaldehyde agarose gel electrophoresis and autoradiography image.

### Northern blot analysis

Accumulation of reporter gene RNA was detected by Northern blot analysis using probe with sequences complementary to R-luc. Briefly, R-luc probes were generated by PCR with primer set (Rluc-F: TCCGCTAGAGCCACCATGAC and Rluc-R: GGCCCTTCACCTTCACGAAC). The PCR amplification conditions were 95 °C for 5 min followed by 30 cycles of denaturation at 95 °C for 1 min, annealing at 55 °C for 2 min, extension at 72 °C for 2 min, and a final extension at 72 °C for 7 min. Radiolabeled DNA probe was generated by random primer labeling with α-[p^32^] dATP.

Total RNA was harvested from cells co-transfected with reporter plasmid pRluc and plasmid expressing wild type (WT) vhs (HSV-1, or PrV), or PrV vhs mutants with deletion of individual boxes by RNeasy^®^ mini kit (QIAGEN). Subsequently, total RNA was separated on a 1.3% denaturing formaldehyde gel and transferred to a Hybond-N^+^ membrane (GE Healthcare Bio-Science Corp., Piscataway, NJ, USA). Following UV-crosslinking fixation and pre-hybridization (for 1 h at 68 °C in pre-hybridization buffer 0.5 M sodium phosphate, 7% SDS and 1 mM EDTA), the membrane was hybridized with radiolabeled DNA probe at 68 °C overnight. After washing steps, the membrane was exposed to a phosphoimage screen (Fuji, Tokyo, Japan) and detected with a Bio-Imaging Analyzer (BAS-2500; Fuji). The relative Rluc RNA level to mock control was plotted.

### Luciferase reporter assays

The effect of PrV vhs on overall translation was evaluated by luciferase reporter assays. Firstly, 293T cells were seeded on the day before transfection at a density of 1 × 10^5^ cells/well in a 24-well plate. 800 ng of vhs plasmids were co-transfected with 800 ng of construct expressing luciferase by Lipofectamine 2000^®^ reagent (Invitrogen) according to the manufacturer’s instructions. Renilla luciferase expression was detected at 24 h post-transfection using a Dual-Glo luciferase assay system (Promega) and measured by FLUOstar OPTIMA microplate reader (BMG Labtech, Offenburg, Germany).

### Statistical analysis

All the results were conducted at least three independent repeats. Data was showed as mean ± SD. Pair *t* test was used to evaluate significant difference. The results of analysis were conducted by the GraphPad Prism5 statistical package analysis tool (GraphPad Software, San Diego, CA, USA). The p value less than 0.05 indicates the statistically significant difference and was shown asterisk sign (*) in all figures.

## Results

### In vitro assay of ribonuclease activity mediated by PrV vhs

Ribonuclease activity of a thioredoxin-PrV vhs fusion protein has been previously described; nevertheless, the vhs assay used involved the degradation of RNA markers in reaction buffer at high concentrations of NaCl (0.3 M) and imidazole (0.25 M) [16]. Taking this into consideration, because the biochemistry properties of PrV vhs-mediated enzyme activity still remains largely unknown, initially the nuclease activity of PrV vhs in the present study was measured following a well-established hydrolysis assay system [8, 18, 24]. To do this, the first step was to express and purify the PrV vhs protein using an *E. coli* expression system. A construct, pET44-PrV vhs, which has a NUS-tag sequence fused to the N-terminus region of PrV vhs was created and the structure of this expression plasmid is shown in Figure 2A. This plasmid was introduced into *E. coli* and expression of the recombinant PrV vhs protein was induced with IPTG (Figure 2B, lane 2). This was followed by purification of the protein using Ni–NTA affinity chromatography (Figure 2B, lane 3). To avoid any unexpected effect on vhs activity caused by the NUS fusion tag, thrombin cleavage was conducted to remove this tag (Figure 2B, lane 4). The identity of PrV vhs was confirmed by Western blot analysis using hexa-histidine antibody (Figure 2C), and also by mass spectrometry (data not shown).

The decay of single stranded (ss) RNA mediated by the PrV vhs protein was analyzed using NUS protein as the negative control. As is indicated in Figure 2D, the RNA was degraded in the presence of PrV vhs, while the same RNA was stable when treated with NUS protein. At 60-min incubation time with the PrV vhs, more than 80% of the RNA present in the assay mixture had been degraded (Figure 2E), which indicates that the purified recombinant PrV vhs, on its own, did indeed harbors ribonuclease activity when using this assay.

### Substrate specificity of PrV vhs mediated nuclease activity

**Figure 3:**
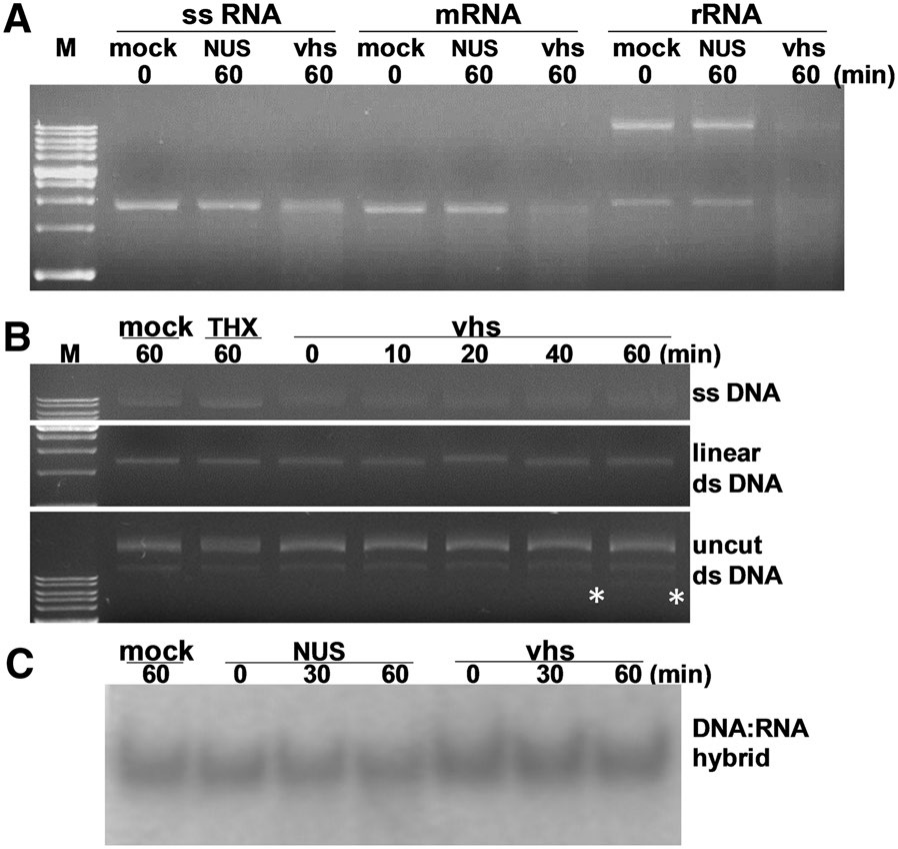
**An in vitro assay of RNase activity mediated by PrV vhs using various substrates.** (**A**) Different types of RNA, including single stranded RNA without cap (ssRNA), with cap and polyA tail (mRNA) and cellular total rRNA, were incubated with assay buffer (mock), recombinant vhs and the appropriate tag proteins, either NUS or Thioredoxin (THX) (negative control), for various times (as indicated above the gel). The degradation pattern was resolved by agarose electrophoresis. In addition to RNA, various DNA substrates (**B**) and a DNA-RNA hybrid (**C**) were also tested.

Sequence alignment has indicated that the N-terminal and internal domains of the vhs protein share similarity with several cellular nucleases including the human Flap endonuclease (FEN-1), bacteriophage T4 RNase H and bacterial DNA polymerase I [24]. The substrates of these nucleases are not limited to ssRNA; hence, the nuclease activity of PrV vhs was investigated using different substrates, namely various form of RNA (ssRNA, rRNA, mRNA), three forms of DNA (ssDNA, linear dsDNA, uncut dsDNA), and a DNA-RNA hybrid; this was carried out in an in vitro system. Similar to HSV vhs, PrV vhs was found to degrade ssRNA and RNA with a cap and poly A tail structure (mRNAs). However, PrV vhs also was able to target rRNA for degradation, which is a significant difference from the activity profile of HSV-1 vhs (Figure 3A). In contrast to the RNA substrates investigated, substrates consisting of DNA, including DNA/RNA hybrid molecules, were resistant to the hydrolytic activity mediated by PrV vhs protein (Figures  3B and C).

### The requirements for cofactors when RNase activity is mediated by PrV vhs

Ribonuclease activity is usually dependent on the presence of ions (Mg^2+^, K^+^) or an energy-generating component (ATP or GTP) [7]. To date, the cofactors required for the PrV vhs-mediated decay has not yet been identified. To remedy this, experiments using a set of buffers consisting of different combinations of catalytic factors (Table 2) were set up to examine the effects of these cofactors on RNA degradation mediated by PrV vhs.

**Table 2:** Various assay buffers used in this study.

	A	B	C	D	E	F
80 mM K^+^	**+**	**+**	**+**	**+**	**−**	**−**
2 mM Mg^2+^	**−**	**−**	**+**	**+**	**+**	**−**
0.25 mM ATP	**−**	**+**	**−**	**+**	**+**	**−**
0.1 mM DTT	**+**	**+**	**+**	**+**	**+**	**+**
1.6 mM Tris–HCl (pH 7.8)	**+**	**+**	**+**	**+**	**+**	**+**

**Figure 4:**
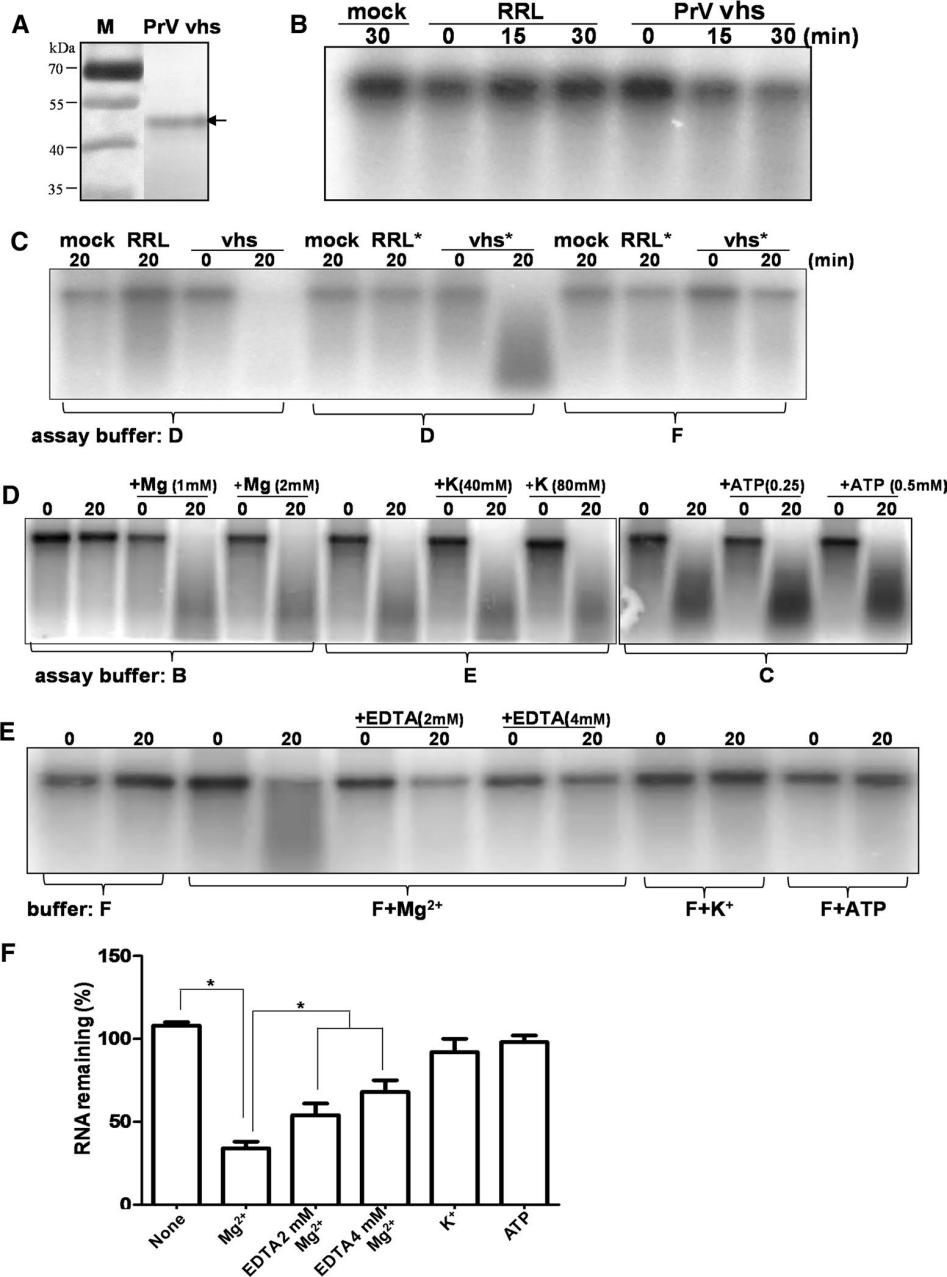
**The catalytic factor requirements for RNase activity mediated by PrV vhs.** To increase the sensitivity, PrV vhs was synthesized using the TNT^®^ T7 Quick coupled Translation system (RRL) and simultaneously labeled with S^35^ (**A**). (**B**) Rabbit reticulocyte (RRL) translated PrV vhs protein displays ribonuclease activity in vitro. RNA internally labeled with α-[P^32^]-ATP was incubated with only assay buffer (mock control), RRL (as a vhs negative control), or in vitro translated PrV vhs at 37 °C for the indicated times (0, 15, and 30 min). The RNA reaction products were resolved by agarose gel electrophoresis followed by autoradiography. (**C**) Contribution of positive ions (e.g. Mg^2+^, K^+^) and ATP to PrV vhs mediated RNase activity. To deplete the residual ions in RRL, desalted plain RRL or RRL translated vhs, as indicated by asterisks, were prepared using Sephadex G-25 spin columns. The RNase activities of the desalted lysates were analyzed in assay buffer D or buffer F containing all, or none of the three factors (Mg^2+^, K^+^, ATP), respectively. To identify the requirements in terms of individual co-factor for vhs-dependent RNase activity, the RNA degradation activity of desalted PrV vhs was further analyzed in two systems: either using buffers missing one of the three co-factors, namely buffers B, E, and C without Mg^2+^, K^+^, or ATP, respectively (**D**), or in buffers containing only one co-factors (buffer F supplemented with Mg^2+^, K^+^, or ATP) (**E**). To confirm the contribution of Mg^2+^, the divalent chelator EDTA was added to the reaction containing Mg^2+^. Nuclease activity was measured using a Kodak image analyzer system and the amounts of RNA remaining (%) of three independent assays were plotted (**F**).

To increase the sensitivity of assay system, PrV vhs protein translated in RRL system (Figure 4A) was incubated with RNA substrates that had been internally labeled with a radioactive isotope α-[p^32^]. As shown by autoradiography in Figure 4B, degradation of RNA was obvious at 15-min post PrV vhs treatment, while RNA incubated with the RRL translation system alone (the mock control) remained unaffected throughout the time of the reaction (Figure 4B).

Next, in order to investigate what ions are necessary for activity, any residual salts carried over from the RRL translation system were depleted from either the RRL translation system itself or RRL translated vhs protein; this was done using a spin column (NAP 5, GE) followed by dialysis against assay buffer containing only Tris–HCl. The results show that, in the buffer deficient with all three catalytic factors (Mg^2+^, K^+^ or ATP), the desalted vhs protein (marked with an asterisk in Figure 4C) was unable to bring about RNA degradation (Figure 4C, buffer F). However, RNA degradation was restored when all three catalytic factors were added back into the assay system (Figure 4C, buffer D).

Subsequently, vhs RNA degradation was determined using three different buffers, B, E, and C, each of which lacked one of the three factors being tested, namely Mg^2+^, K^+^, or ATP, respectively. A lack of K^+^ or ATP alone did not affect the RNase activity of PrV vhs (Figure 4D, buffer E, and C, respectively). However, RNase activity was lost when Mg^2+^ was absent (Figure 4D, buffer B, lane 2), while supplementation with magnesium ions (+Mg 1 or 2 mM) restored the ability to degrade RNA (Figure 4D, buffer B, lane 4, 6). These findings indicate the importance of Mg^2+^ to PrV vhs activity.

Finally, assay buffers G, H, I, which contain only Mg^2+^, K^+^, or ATP, respectively, were used to explore whether any of these factors alone is sufficient in themselves for PrV vhs-dependent RNase activity. RNA degradation activity by desalted PrV vhs was not observed in buffer F without any of the catalytic factors (Figure 4E, buffer F). The presence of Mg^2+^ alone was sufficient for RNase activity mediated by PrV vhs (Figure 4E, buffer F+ Mg^2+^), but no such effect was found for K^+^ or ATP alone (Figure 4E, buffer F+ K^+^, F+ ATP, respectively).

Furthermore, addition of EDTA, a divalent cation chelator, significantly inhibited the catalytic ability of PrV vhs in the presence of Mg^2+^ (Figure 4E, +EDTA 2 or 4 mM). Overall, when the RNA remaining (%) under the various different assay conditions was compared, the results indicated that Mg^2+^ alone was able to allow the degradation of RNA substrates by PrV vhs, while, K^+^ and ATP alone were not sufficient for activity (Figure 4F).

### The contribution to RNase activity of the four highly conserved functional boxes and the fourteen conserved residues found in PrV vhs

**Figure 5:**
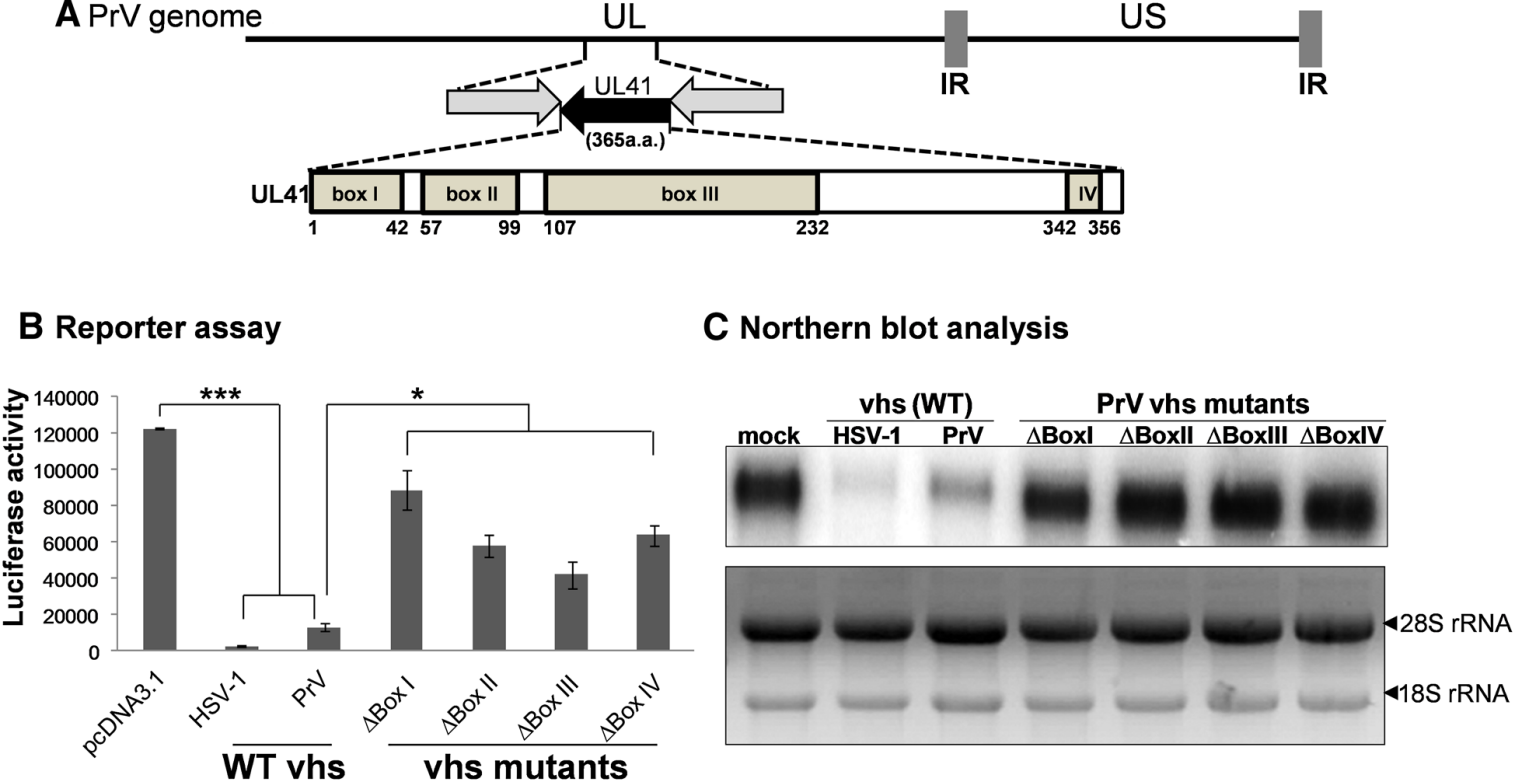
**The contribution of conserved functional domains to vhs-dependent RNase activity.** The locations of the four boxes (I–IV) in PRV vhs are presented (**A**). Constructs with deletions of one of the PrV vhs boxes were generated and co-transfected with plasmid expressing luciferase in human 293T cells. Cells transfected with empty vector (pcDNA3.1) or HSV-1 vhs (HSV-1) served as negative and positive controls, respectively. The effects of the individual boxes were evaluated using this in vivo system (luciferase reporter assay). The luciferase activities of three independent assays were plotted (**B**). In addition, the RNA level of the reporter gene was also monitored by Northern blot analysis (upper panel in **C**). A loading of total RNA of 10 μg per gel lane was used.

Based on the sequences alignment of the vhs proteins of the various *α*-*herpesviruses*, four conserved functional domains can be identified (Figure 1) and the location of these boxes on PrV vhs are illustrated in Figure 5A. Up to now the contribution of these four boxes to PrV vhs-induced RNase activity has remained unclear. Therefore, an in vitro a reporter assay, modified from a previous study [25], was set up to determine PrV vhs-dependent ribonuclease activity using six constructs. The constructs express the wild type vhs protein of HSV-1 as a positive control, the wild-type PrV as a second control and the four proteins with individual deletions of the four conserved boxes within PrV vhs. The effects of these deletions of the vhs protein on RNA degradation were evaluated by co-transfected each construct with a luciferase reporter plasmid in order to measure the loss of luciferase mRNA.

Luciferase activity in cells co-expressing the wild-type viral vhs proteins from either HSV-1 or PrV was significantly lower than the luciferase activity in the control cells transfected with an empty expression vector (pcDNA3.1) (Figure 5B). The decrease in luciferase activity was correlated with the amount of reporter RNA present in the transfected cells based on Northern blot analysis (Figure 5C). When the four vhs mutants were compared to the wild type PrV in this system, both reporter enzyme activity and RNA level were greatly elevated compared to the cells expressing wild-type vhs. These findings indicate that all four predicted conserved boxes seem to contribute to PrV vhs-dependent RNA degradation.

### Sequence alignment and the effect of mutation of various highly conserved residues within the PrV vhs protein on RNase activity

The specific sequences that contribute to the RNase activity of HSV-1 vhs have been identified. However, in this regard, little is known about PrV vhs. Despite the difference in size of the two proteins (489 vs. 365 residues) and the low sequence similarity between HSV-1 vhs and PrV vhs (Figure 1), several functionally significant residues located within the active site (Boxes I-III) are conserved in PrV vhs (as indicated by the arrowheads in Figure 1), including D34, D82, K96, E192, D194, T211, D213, D215, and D261, as well as amino acid 214. The first seven key amino acids have been shown to be critical to both nuclease activity in general and to the ribonuclease activity of HSV-1 vhs (as summarized in Table 3); this was determined using a combination of genetic and biochemical approaches [24]. Importantly, residue 214 is a vhs activity determinant and a HSV-1 mutant (vhs1) harboring a point mutation at residue 214 (Thr214 → Ile) in the UL41 coding region results in complete abolishment of vhs activity [26, 27]. The coordinate position of T214 on PrV vhs is residue T172.

**Table 3:** Impact of the fourteen highly conserved residues on activity of HSV-1 vhs protein and other nucleases.

Residues corresponding to PrV vhs	Corresponding residues									
	HSV vhs					T4 RNase H			Human FEN-1	
		RNase activity [13]	vhs activity [24]	eIF4H binding [13]		Mg^2+^ binding [31, 32]	Nuclease /substrate binding [23, 35, 36]		Mg^2+^ binding [32, 34, 37]	Nuclease /substrate binding [33]
D34	D34	−	−	+	D19	−	−	D34	−	−
D81	D82	−	−	+	D71	−	−	D86	−	−
K95	K96	−	−	N	K87	N	−	R103	N	+
E150	E192	−	−	+	E130	N	N	E158	−	−
D152	D194	−	−	+	D132	−	−	E160	−	N
D169	T211	−	<	−	S153	−	<	T177	N	N
D171	D213	−	−	+	D155	−	−	D179	−	+
D172	T214	−	−	−	D156	N	N	D180	N	N
D173	D215	−	−	+	D157	−	≪	D181	−	∓
D219	D261	−	<	+	D200	−	+	D233	−	−
P343	P465	N	N	−	−	N	N	E357	N	N
P345	P467	N	N	−	−	N	N	E359	N	N
L352	L474	N	N	−	−	N	N	K366	N	N
W356	W478	N	N	−	−	N	N	G370	N	N

“−” indicates the point mutations lost RNase activity and the ability of Mg^2+^ or eIF4H binding.

“<” indicates the point mutations partially reduced RNase activity and the ability of Mg^2+^ binding.

“N” indicates the impact of those residues have not yet been defined.

∓ indicates FEN-1 D181 mutation affects their ability of ribonucleases, but does not affect its ability to bind with the substrate.

It is worth noting that the above predicted functional important residues are located near the N-terminal (residues 1–103 within Box I and Box II) and within an internal region (residues 165–265 within Box III) of the HSV-1 vhs coding region (Figure 1). Furthermore, in addition to these positions, an additional four amino acids (P343, P345, L352 and W356), which are found in functional Box IV, are conserved across members of *α*-*herpesviruses*, including HSV-1, HSV-2 and PrV. To define the contribution of all of the above residues to PrV activity, we altered each of the corresponding fourteen residues in the PrV vhs (D34, D81, K95, E150, D152, D169, D171, D172, D173, D219, P343, P345, L352, and W356) to alanine by site-directed mutagenesis using the primers listed in Table 1.

**Figure 6:**
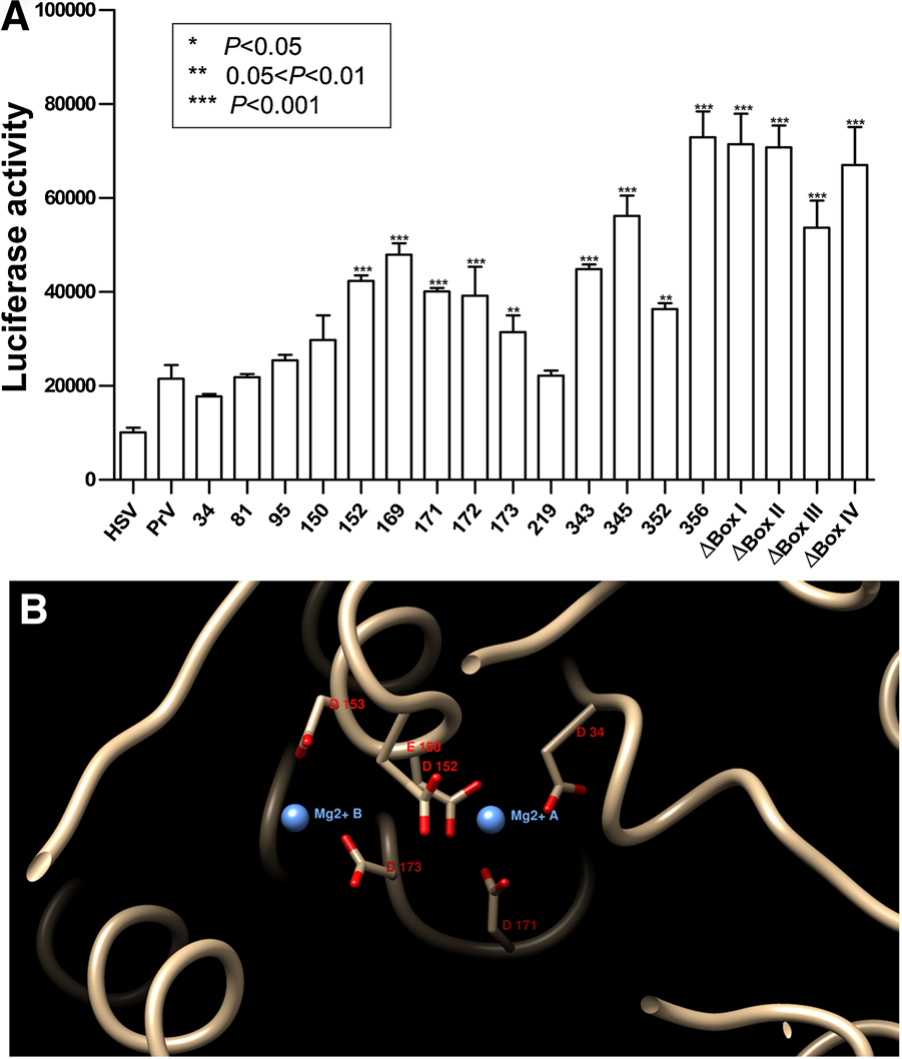
**Contribution of the fourteen conserved residues present in PrV vhs to RNase activity in vivo.** Reporter plasmid, pRluc (encoding Renilla luciferase) was co-transfected with each one of the vhs constructs into human 293T cells. The effect of each vhs protein on reporter gene expression was evaluated based on luciferase activity. HSV-1 vhs (HSV) served as a positive control for PrV vhs-dependent RNase activity. The luciferase activities of three independent assays were plotted (**A**). A structure indicating the key residues of PrV vhs surrounding the Mg^2+^ in the protein was created by simulation (**B**).

The effect of these mutations on PrV vhs activity was then investigated by Luciferase reporter assay. Among these fourteen conserved residues, mutations at residues 34, 81, 95, 150, and 219 did not affect the ribonuclease activity mediated by PrV vhs (Figure 6A); while point mutation at D152, D169, D171, D172, D173, P343, P345, L352, and W356 did significantly reduce the vhs activity detected.

## Discussion

To date, the HSV-1 vhs protein has been well characterized, however little is known about the bioactivity of the PrV vhs protein. Previously, the RNase activity of PrV vhs was identified using standard RNA markers, and a recombinant vhs-thioredoxin fusion protein in buffer containing very high concentrations of imidazole and NaCl. This implies that there might be problems associated with the possible effects of salt, the bulky thioredoxin tag (with size of ~20 kDa) and intrinsic RNase activity contamination. Furthermore, cofactor requirements, such as Mg^2+^ and/or ATP, were not considered [16]. This motivated us to further characterize the ribonuclease activity of PrV vhs in more depth.

Accumulating evidence has suggested that the vhs protein serves as an endoribonuclease or a component of a ribonuclease complex [9, 11, 12]. To define the role of vhs, a soluble protein purified to homogeneity is necessary; however this was jeopardized by the poor solubility of native vhs. In 2006, HSV vhs itself was proved to be an RNase for the first time using GST-tagged vhs purified under native condition with a lower concentration of detergent [28]. In a similar manner, in order to avoid the insolubility issue, PrV vhs was expressed as a fusion protein with a NUS-tag in the present study. As shown in Figure 2B, the improved solubility and yield (lane 2) allowed purification of the recombinant PrV vhs protein to a high homogeneity with ease (lane 3), and thereby residual RNase contamination from *E. coli* was minimized. Since, addition of a fusion peptide can be detrimental to vhs RNase activity, the NUS tag (~70 kDa), which was added at the N-terminal of the recombinant PrV vhs, was removed as part of the present study. The RNase activity of PrV vhs was confirmed by an in vitro RNA hydrolytic assay following the method established by other research groups [17, 18]. To further exclude the possible contamination with unwanted RNase activity from proteins expressed in *E. coli* or other sources, the RNase inhibitor, RNaseOUT^®^, was constantly included in the assay, and vhs translated using the RRL system was also used in this study. It is worth noting that the vhs-dependent RNase is resistant to this RNase inhibitor [7, 9, 12, 18], while another study using recombinant GST-vhs expressed from *E. coli* indicated that HSV-1 vhs nuclease activity is blocked by an RNase inhibitor [28]. Using the PrV vhs, significant RNase activity was observed in the presence of an RNase inhibitor, indicating that activity of PrV vhs is not affected by the RNase inhibitor.

The ribonuclease activity of PrV vhs resembles that of HSV vhs; nevertheless, the biochemical characteristics of these two proteins are not identical. They both require Mg^2+^ and target mRNA for degradation. Lu et al., reported that the vhs of HSV-1 produced from yeast displayed little if any vhs-dependent RNase activity and seemed to require one or more mammalian cellular factors for efficient activity [9]. In our study, the purified recombinant PrV vhs protein did exhibit RNase activity without the aid of other proteins. Moreover, unlike HSV-1 vhs, PrV vhs is able to degrade not only mRNA, but also rRNA and ss RNA without a cap and polyA tail using an in vitro assay system. Of note, in the reporter assay, rRNA level was not significantly affected by PrV vhs in transfected cells (as shown on the agarose gel in Figure 5C). Reporter assay resembles to an in vivo scenario in which rRNAs are associated with a number of ribosomal proteins, while ribonuclease assay is an in vitro system of which rRNA substrate was extracted by phenol-based reagent that degrades proteins and renders rRNA unprotected. Hence, rRNA with or without cellular protein association is likely the cause of ambiguous findings in two systems.

The amino acid sequences of vhs orthologs of *alphaherpesviruses* share similarity with a number of nucleases from various organisms, including flap endonuclease 1 (FEN-1) and RNase H [24]. FEN-1 is a structure-specific nuclease that participates in DNA metabolic pathways [29], and RNase H removes RNA in a DNA/RNA duplex substrate. Stevens demonstrated that, in addition to DNA, the FEN-1 nuclease also is able to cleave a cap-independent RNA substrate at 20% of the rate of DNA substrates when Mg^2+^ is present [30]. Based on these similarities, an attempt was made to explore whether PrV vhs is able to function as a DNase. It appears that various DNA molecules, including both single stranded and double stranded forms, are not a substrate of PrV vhs (Figure 3B). Notably, when uncut plasmid was included in the assay, compared with the control group, incubation of PrV vhs yields an extra banding with faster mobility (indicated by asterisks). However, whether it is a cleaved fragment of dsDNA or another type of dsDNA entity with a different conformation that is generated by the action of PrV vhs requires further investigation.

It is obvious that, within the highly homologous regions of the vhs protein, there are several active-site residues of nucleases that are conserved (Table 3). In current study, amino acids 152,169,171,172,173, 343, 345, 352 and 356 were found to be important for PrV vhs ribonuclease activity (Figure 6A). Among these residues, PrV vhs T172, which corresponds to T214, was identified as a key activity determinant of HSV-1 vhs based on the findings obtaining with a HSV-1 mutant virus, vhs1 [26, 27]. D34, D82, K96, E192, D194, T211, D213, D215 and D261 are conserved functional residues in HSV-1 vhs, and it has been shown that changing these abrogates vhs functioning [12, 13, 23, 24, 31–36]. However, not all of these amino acids are critical to PrV vhs functionality and mutations of only four residues, namely. D152, T169, D171, and D173, reduced vhs activity in an in vivo reporter assay. Structure simulation suggests that D152, T169, D171, and D173 may be located either in the active site within the protein or in close proximity to the binding site for the catalytic metal ions (Mg^2+^), both of which are very important to vhs-induced RNase activity (Figure 6B).

It is worth noting that the nine key amino acids believed to be related to HSV-1 vhs dependent RNase activity were initially predicted based on the structure similarity of vhs with other cellular nuclease [24], and are found in the N-terminal and internal region of HSV-1 vhs. This means that the impact on vhs activity of residues located in C-terminus has not been explored up to now. In the present study we have shown that four additional amino acids (P343, P345, L352 and W356) that are found within the predicted Box IV are completely conserved across members of α-herpesviruses (HSV-1, HSV-2, VZV, EHV, PrV) [15]. When a similar analysis to the other conserved amino acids was carried out on these amino acids, it was found that these also contribute to PrV vhs activity. Previous studies have demonstrated that the C-terminus of HSV vhs is involved in the interaction of vhs with eIF4H [12]. Page and Read identified that a deletion of as few as 108 amino acids from the C terminus of HSV-1 vhs is sufficient to hinder vhs binding to eIF4H, but not to eIF4A II [13]. Hence, it is possible that mutants at residues 343, 345, 352 and 356 in PrV vhs obstruct interaction of the protein with other cellular factors (e.g. eIF4H) and consequently this affects RNA degradation activity. Nevertheless, whether these cellular factors play an important role in PrV vhs activity remains unclear and requires further investigation.

In conclusion, we have characterized the nuclease activity of the PrV vhs protein using both in vitro and in vivo systems. The enzyme is similar to, but not identical to, HSV-1 vhs. The activity of PrV vhs requires Mg^2+^, but does not require either K^+^ or ATP. The enzyme is resistant to an RNase inhibitor. Furthermore, the enzyme degrades not only mRNA, but also rRNA. Despite the low sequence similarity between PrV vhs and other vhs proteins, some, but not all, of the conserved residues critical for HSV-1 vhs RNase activity are essential for PrV vhs RNase activity.
